# Slipped capital femoral epiphysis: an ultrastructural study before and after osteosynthesis

**DOI:** 10.3109/17453674.2010.483987

**Published:** 2010-05-21

**Authors:** Francesco Falciglia, Angelo Gabriele Aulisa, Marco Giordano, Renata Boldrini, Vincenzo Guzzanti

**Affiliations:** ^1^Orthopaedics and Traumatology Division, Institute of Scientific Research, Bambino Gesù Children's HospitalRome; ^2^Department of Pathology, Bambino Gesù Children's HospitalRome; ^3^University of Cassino, CassinoItaly

## Abstract

**Background and purpose:**

Several different theories have been proposed to explain the pathogenesis of slipped capital femoral epiphysis (SCFE). Using transmission electron microscopy (TEM), we carried out an ultrastructural study of core biopsy specimens of the physis at various stages of the disease.

**Methods:**

Core biopsies were performed in 6 patients with different forms of SCFE during the first operation, and in 3 of them when removing the osteosynthesis material before physeal closure. The specimens were prepared for TEM examination.

**Results:**

In 6 specimens obtained at first surgery, a marked distortion of the physeal architecture was observed. In 2 of the 3 specimens obtained at removal of the osteosynthesis material, the physis showed a more normal organization.

**Interpretation:**

The improvement of the pathological alterations observed in the 2 cases after surgical intervention leads us to consider the possibility that when the growth plate is stabilized directly by pinning or indirectly by creating more optimal loading conditions with an intertrochanteric osteotomy, the morpho-functional characteristics of the physis can be restored and its growth process may resume.

## Introduction

The pathogenesis of SCFE remains unclear and several different theories have been proposed (Harris 1950, Bois et al. 1963, Renne 1967, Chung et al. 1976, Eisenstein and Rotschild 1976, Oka et al. 1979). Various authors have reported histological alterations of the growth plate in slipped capital femoral epiphysis (SCFE) (Kleinberg and Buchman 1936, Howorth 1941, 1949, Harris 1950, Lacroix and Verbrugge 1951, Ponseti and McClintock 1956, Wagner 1961, Taillard et al. 1964, Consolo and Randelli 1971, Ippolito et al. 1981, Agamanolis et al. 1985a, Portigliatti Barbos et al. 1985, Guzzanti et al. 2003). To our knowledge, however, there have only been 3 ultrastructural studies that have considered the pathology of the slippage in SCFE (Mickelson et al. 1977, Agamanolis et al. 1985b, Ippolito et al. 1989).

In the literature, there is agreement concerning the deficiency and disarray of collagen fibrils in the matrix, but controversy with regard to interpretation of the architecture and the morphological alterations of the proteoglycans (PGs). Ippolito et al (1989) found the characteristic alterations of the growth plate in 2 cases of pre-slipping; they believed that the pathology is present before the slippage occurs and that it may have been caused by a change in chondrocyte metabolism. The consequent modifications of the matrix, due to altered synthesis of collagen and PGs, would predispose to slippage. Other authors (Mickelson et al. 1977, Agamanolis et al. 1985b) did not find modifications of PGs: they believed that the alteration of the growth plate is due to a metabolic factor (with consequent deficiency and disarray of collagen fibrils) and to a mechanical factor.

The aim of the present study was to help clarify the anatomical-pathological aspects and pathogenesis of SCFE. We performed an ultrastructural study of the growth plate during the different periods with various manifestations of the disease (stable/unstable with different degrees of slipping: mild, moderate, severe) and, in some cases, during the surgical intervention period before physeal closure. (To our knowledge, there has been no previous ultrastructural study of the growth plate performed after osteosynthesis before physeal closure).

## Patients and methods

**Figure 1.**
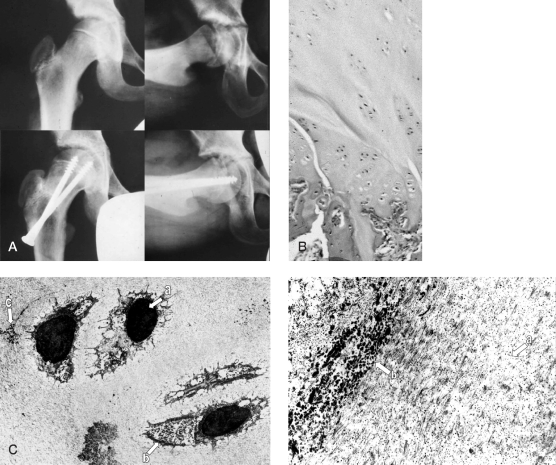
The growth plate of slipped capital femoral epiphysis in a case of mild unstable slipping (case number 3 in the Table). A. Before and after the operation when biopsy was performed. B. Semi-thin section, light microscopy. The chondrocytes of the resting zone are grouped in small clusters while in the proliferating and hypertrophic zone, chondrocytes are arranged in large clusters separated by thin metachromatic septae or by areas of non-metachromatic extracellular matrix (toluidine blue, ×200). C. Ultra-thin section, TEM. The chondrocytes in the proliferative and hypertrophic zones (obtained from the semi-thin section in B) are arranged in clusters. The cells show an increase in nuclear density (arrow a) and cytoplasmatic density (arrow b). Matrix vesicles and cellular debris are present around the cells (arrow c) (× 4,500). D. Ultra-thin section, TEM. Matrix of the hypertrophic zone of semi-thin section in B. The abnormal matrix is loosely arranged and the fibrils are slightly thinner (arrow a); in some areas they are 20–30 nm thick (normal: 80–120 nm thick) (Agamanolis et al. 1985b). Abundant matrix vesicles and cellular debris are present (arrow b) (× 8,000).

Core biopsies of the chondroepiphysis were performed in 6 children with unilateral SCFE (4 of them boys). The mean age was 11 (10–14) years. Informed consent was obtained from the parents of all 6 children. The SCFEs were graded by the magnitude of the slip (in the lateral view) according to Guzzanti and Falciglia (1991), as mild (< 30°), moderate (30°–50°), or severe (>50°) and they were classified as being unstable type (unable to bear weight on the affected leg with or without support) or stable type (able to bear weight on the affected leg with or without support) (Loder et al. 1993) (Table). In all 6 cases, core biopsies of the chondroepiphysis were obtained at initial surgery (Figure 1).

**Table:** 

A	B	C	D	E	F	G	H	I	J	K	L	M	N
1	M	12, 5	3 months	Stable	0–10°	L	DSF	CN	/	/		8	No
2	F	9, 6	4 months	Stable	0–10°	R	DSF	CN	/	/		10	No
3	F	13, 5	2 days to 2 months	Unstable	20°	R	DSF	CN	/	/		9	No
4	M	10	10 months	Stable	60°	R	LIO	CN	12	24	A	28	No
5	M	10, 6	8 months	Stable	45°	L	LIO	CN	11, 8	14	B	32	No
6	M	12	3 days to 4 months	Unstable	25°	R	Pinning	CN	13, 4	16	B	24	No

Symptoms in cases 3 and 6 were acute-chronic. A Case B Sex C Age expressed in years, months. D Time of symptoms E Type F Degree of slipping G Side H Operation method DSF Double screw fixation LIO Linear intertrochanteric osteotomy without physeal involvement I Ultrastructural observation CN Cellular necrosis in hypertrophic zone, collagen fibrils altered J Age at removal expressed in years, months. K Time after initial operation expressed in months. L Ultrastructural after removal before physeal closure A Collagen fibrils alteration, disarray of chondrocyte columns B Normal banded collagen fibrils and chondrocytes into columns M Time of physeal closure from operation in months. N Further slipping

In cases 4–6, biopsies were also obtained at the time of hardware removal from the osteosynthesis, done before physeal closure. 2 patients (numbers 4 and 5 in the Table, Figure 3A) suffered from continuous bursitis near the nail of Smith Petersen and plate of McLaughlin used for linear intertrochanteric osteotomy without causing premature closure of the physis (Fineschi and Guzzanti 1986). In the third patient (number 6 in the Table), who was affected by mild-unstable SCFE, there was a growing-away phenomenon of the epiphysis during treatment with in situ pinning (the helicoids of the screw did not anchor to the epiphysis with all 4 helicoids inserted at the first operation, but only with 2). In this 12 year-old boy, the screw was removed 16 months after osteosynthesis and a new screw fixation was done.

Core biopsies were obtained using a Jamshidi needle (4-inch (10.1-cm), 8-gauge; Baxter Healthcare Corp., Deerfield, IL) prior to drilling in the planned track of the screw or the nail. In cases 4–6, the core biopsies at the time of removal of the osteosynthesis material were obtained making a new track. All biopsy specimens were cylindrical and were 2.5 cm in length and 0.25 cm in diameter. The specimens were fixed in 4% glutaraldehyde in 1 M phosphate buffer (pH 7.4) at 4°C for 12 h.

Subsequently, they were reduced into smaller sections by stereomicroscopy and post-fixed with Millonig's buffer in 1.3% osmium tetroxide (pH 7.4) for 1 h, dehydrated in a graded series of alcohol and thus reduced, and they were then enclosed in epossidic resin (agar 100) (Bozzola and Russel 1992).

**Figure 2.**
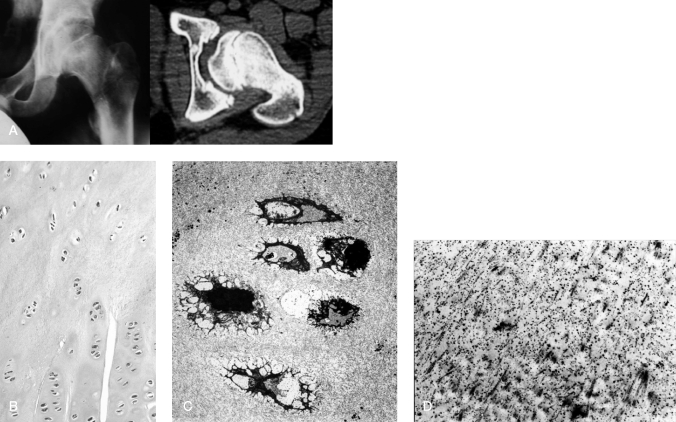
The growth plate of SCFE in a case of stable moderate slippage (case number 5 in the Table). A. Just before operation. B. Semi-thin section, light microscopy. The chondrocytes in the proliferating and hyperthrophic zone show an irregular organization in columns with gradual loss of longitudinal septa (toluidine blue, ×200). C. Ultra-thin section, TEM. The chondrocytes in the proliferative and hypertrophic zones (obtained from the semi-thin section in B) are arranged in large clusters (× 4,500). D. Ultra-thin section, TEM. In the hypertrophic zone, the extracellular matrix separating the clusters of chondrocytes contain thin, unbanded collagen fibrils with haphazard orientation; matrix vesicles and cellular debris are present (×8,000).

**Figure 3.**
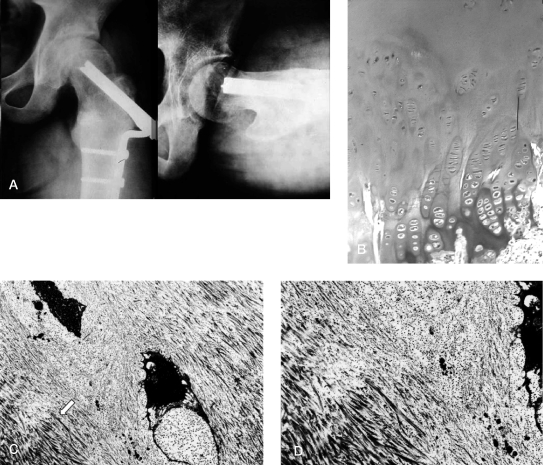
The growth plate of SCFE in a case of stable moderate slippage (case number 5 in the Table, the same case as in Figure 2). A. Before removal of the nail and plate and performance of core biopsy of the still open growth plate at age 12. B. Semi-thin section, light microscopy. The chondrocytes in the proliferating and hyperthrophic zone show a regular organization in columns with transversal and longitudinal septa evident (toluidine blue, ×200). C. Ultra-thin section, TEM. The chondrocytes in the proliferative and hypertrophic zones (obtained from the semi-thin section in B) are arranged into columns, separated by normal longitudinal septa (white arrow) (×3,000). D. Ultra-thin section, TEM. Detail of C. The extracellular matrix appears normal and the collagen fibrils show normal thickness (80–120 nm), are banded, and are better orientated (×6,000).

Semi-thin sections were stained with toluidine blue for light microscopy in order to choose the areas for ultra-thin sectioning (Figures 1, 2, and 3).

The ultra-thin sections (of 80–90 nm) were obtained using an ultramicrotome with diamond blade.

The ultra-thin sections were mounted on a copper/rhodium (Cu/Rh) grid (200 square mesh; Electron Microscopy Sciences, Fort Washington, PA), stained with uranyl acetate and lead citrate, and examined by transmission electron microscopy (TEM).

## Results

In all specimens obtained at osteosynthesis, it was possible to observe some common characteristics. The growth plates showed a marked distortion of the architecture as well as disorganization and disarray of chondrocyte columns that were arranged in large clusters, particularly in the proliferative and hypertrophic zones (Figures 1 and 2). Some cells were proliferative, and others were necrobiotic. Chondrocytes were generally smaller and often showed an increase in nuclear and cytoplasmatic density, often obliterating all subcellular details compared to descriptions by other authors (Brighton et al. 1973, Brighton 1978, Agamanolis et al. 1985b) (Figures 1 and 2). There were clear signs of plasma membrane fragmentation, an abundance of residues of membrane in the matrix, and other debris and vesicles. The vital chondrocytes showed rough endoplasmic reticulum (RER), mitochondria, and cisternae of normal appearance. Accumulations of cytoplasmic glycogen were evident at all levels. The matrix showed a marked reduction of collagen both in the extraterritorial matrix and in the longitudinal septa of the proliferative and hypertrophic zones. Collagen fibrils were haphazardly oriented, were not banded, and were 20–30 nm thick—which is thinner than normal: 80–120 nm (Agamanolis et al. 1985b) (Figures 1 and 2). In some areas of the matrix, collagen showed an increase in density while in other areas it was more spaced out. The amount of proteoglycans (PGs) appeared normal. In some areas, we also observed a reduction in PGs and a separation between these and collagen fibrils, but this last observation could have been due to technical artifacts.

The specimens obtained at removal of the osteosynthetic material before physeal closure (numbers 4–6 in Table) showed a better organization of the cells in 2 cases (numbers 5 and 6), with the matrix tending toward the normal, especially in the proliferative and hypertrophic zones (Figure 3). In another case, however, (number 4) there was persistence of the pathological alterations previously described.

In the improved cases, proliferative cells increased and necrobiosis decreased, accompanied by a restoration of the chondrocytes in columns. In the extracellular matrix and the longitudinal septa, the collagen was made up of better-oriented banded fibrils of normal thickness (ranging from 80 to 120 nm in diameter) and was enmeshed in a network of branching PGs (Figure 3).

## Discussion

Analysis of ultrastructural observations allows one to detect the presence of matrix and cellular modifications, even in the initial phases of SCFE disease (mild stable cases). We observed that some cells degenerate and die in zones in which they would be expected to proliferate and hypertrophy. The morpho-functional activity of the growth plate is altered, and it seems to be—in accordance with Agamanolis et al. (1985b)—that the normal process of degeneration is accelerated.

The chondrocytes of the hypertrophic and proliferative zones appear to have lost the capacity to become organized into columns. The collagen fibrils were thinner, unbanded, and showed haphazard orientation in the longitudinal septa.

Our observations concerning the collagenous framework of the growth plate agree with the findings of Mickelson et al. (1977) and Agamanolis et al. (1985b) who concluded that slipping is due to a defect in collagen production by chondrocytes. According to these authors, the sparse, thin, and disoriented fibrils are probably the result of an alteration of the collagen, which is sufficiently severe to cause lack of definition of the longitudinal septa. The different arrangement of collagen fibrils in various zones of the matrix (reactive-regressive change) is probably secondary to mechanical factors. In the PGs, another component of the extracellular matrix, we did not find any abnormalities—either in the morphology or in the distribution. Ippolito et al. (1989) believed that the floccular electron-dense material present in the chondrocytes and in the matrix is caused by an altered production of PGs. However, our observations did not confirm this supposition. In some zones of the growth plate, and more evident in unstable forms of SCFE, PG particles were sparse and widely spaced. This disposition caused the matrix to assume a weak appearance in some areas. In other zones, however, the PGs had mostly increased in thickness. The cause of the modifications in PGs is still uncertain, because of the possibility of extraction artifacts and other variables in the preparation of specimens for TEM examination.

After mechanical stabilization of the growth plate, we observed an improvement in organization and a reduction of necrobiotic cells in 2 cases. The improvement of morphological alterations of the growth plate during treatment may have been due to both biological and mechanical factors. The biological factors, which may cause the slippage, are probably transitory and stabilization (reduction of mechanical factors) of the growth plate may help the recovery process. In the third case, the pathological alterations persisted; this case was the only one with severe slippage (60°).

In conclusion, in accordance with the work of Mickelson et al. (1977) and of Agamanolis et al. (1985b), our observations suggest that slippage of the growth plate is due to weakness of the supporting fibrous network caused by collagen deficiency.
